# Health system efficiency and democracy: A public choice perspective

**DOI:** 10.1371/journal.pone.0256737

**Published:** 2021-09-07

**Authors:** Martin Roessler, Jochen Schmitt

**Affiliations:** Zentrum für Evidenzbasierte Gesundheitsversorgung, Universitätsklinikum und Medizinische Fakultät Carl Gustav Carus an der Technischen Universität Dresden, Dresden, Germany; University of Western Australia, AUSTRALIA

## Abstract

Due to increasing demand and scarce financial resources for healthcare, health system efficiency has become a major topic in political and scientific debates. While previous studies investigating determinants of health system efficiency focused primarily on economic and social influence factors, the role of the political regime has been neglected. In addition, there is a lack of formal theoretical work on this specific topic, which ensures transparency and logical consistency of arguments and implications. Using a public choice approach, this paper provides a rigorous theoretical and empirical investigation of the relationships between health system efficiency and political institutions. We develop a simple principal-agent model describing the behavior of a government with respect to investments in population health under different political regimes. The main implication of the theoretical model is that governments under more democratic regimes put more effort in reducing embezzlement of health expenditure than non-democratic regimes. Accordingly, democratic countries are predicted to have more efficient health systems than non-democratic countries. We test this hypothesis based on a broad dataset including 158 countries over the period 1995-2015. The empirical results clearly support the implications of the theoretical model and withstand several robustness checks, including the use of alternative indicators for population health and democracy and estimations accounting for endogeneity. The empirical results also indicate that the effect of democracy on health system efficiency is more pronounced in countries with higher income levels. From a policy perspective, we discuss the implications of our findings in the context of health development assistance.

## 1 Introduction

The key role of population health in many areas, including social security [1, 2] and economic development [3–5], makes a well-functioning and effective health system one of the most important elements of modern societies. From a global perspective, the extension of healthcare was associated with substantial improvements in core health outcomes like infant mortality rates and life expectancy over the last decades [6]. At the same time, many national health systems face an increasing financial burden [7], which threatens the sustainability of healthcare provision.

Against that background, the efficiency of health systems has become a major topic in scientific and political debates [8]. Basically, health system efficiency refers to the relation between health system inputs (consumed resources) and outputs or outcomes [9]. While outputs refer to “units of activity produced by combining health care inputs” (e.g. surgical procedures, episodes of care, etc.), outcomes are defined as “valued health care outputs” (e.g. quality-adjusted life years or patient reported outcome measures) [9]. Following this definition, a health system becomes more efficient if it increases its output/improves outcomes with fixed amounts of inputs or produces the same output/outcomes with reduced inputs. In the view of growing demand and scarce financial resources for health, ensuring and fostering efficiency therefore is a main policy objective.

Notably, in this study we focus on health system efficiency in terms of outcomes/the population’s health status because of its relevance for the utility of citizens and, presumably, their attitudes towards the government. This efficiency does not necessarily coincide with efficiency in terms of physical output/patient volume [10, 11], which could be evaluated separately.

Previous research has focused on estimating and comparing health system efficiency between countries and over time [12–14]. In addition, determinants of health system efficiency have been investigated in empirical studies [15–17]. However, while most of these studies focus on economic, social, or governance-related covariates of health system efficiency, the role of a countries’ political regime has been neglected. In addition, most empirical studies lack a clear theoretical foundation that could ensure logically consistent identification of mechanisms and impacts of relevant determinants of efficiency.

Using a public choice approach, this study aims to close these gaps by providing a rigorous theoretical and empirical analysis of the relationships between health system efficiency and political regimes. We define a political regime as the entirety of a state’s political institutions, such as elections, the constitution, parliaments, and courts. While ideal democracies are characterized by open, fair, and free elections and a system of checks and balances, those institutions are missing in autocracies, which are often ruled by a small elite [18]. Political and economic impacts of different political regimes have long been analyzed in the public choice literature [19–22].

Public choice may be defined as “the economic study of nonmarket decision-making” [23]. Public choice models generally rely on the assumption of egoistic, utility-maximizing behavior to explain decisions made by relevant agents and their interactions. In this spirit, we combine arguments from studies on health system performance and the public choice literature to develop a simple formal model describing the behavior of a government with respect to investments in population health. Our “public choice model of health system efficiency” indicates that governments under democratic political regimes generally do not choose higher investments in population health but put more effort in eliminating inefficiencies than non-democratic governments. Hence, the model predicts the health systems of democratic countries to be more efficient than the health systems of non-democratic countries. We test this hypothesis based on a stochastic frontier model, whose econometric specification is derived directly from the theoretical model. The estimation results for different population health and democracy indicators support the theoretical implications and the underlying mechanisms.

## 2 A glance at the literature

The performance and efficiency of health systems has been investigated in several studies from an international perspective. While Evans et al. find that health system efficiency in a sample of 191 countries between 1993–1997 varied between nearly fully inefficient to nearly fully efficient [24], the results of more recent studies indicate relatively high average efficiency levels of 80% or more [14, 15]. Variations in health system efficiency were also investigated for subsets of countries [12, 25, 26] or even at the provincial level [27]. Generally, all these studies reveal relevant scope for better health system performance due to improvements of efficiency.

Multiple studies exploring covariates of health system efficiency find that higher levels of economic development as reflected in higher per capita income and per capita health expenditure are associated with higher efficiency [12, 15, 24]. There is also some evidence that countries with more concentrated populations as reflected in higher population density and urbanization have more efficient health systems [12, 14]. In contrast, evidence on the role of funding sources is heterogeneous. Chai et al. find that higher out-of-pocket health expenditure was related to significantly lower efficiency in Chinese provinces [27]. Grosskopf et al. find evidence for weak correlation between health system performance and the share of public funding in the healthcare sector [28]. With respect to hospital sectors in OECD countries, another study reports that public and private funding sources do not differ in their relationship to efficiency [17]. Due to the absence of compelling evidence for relevant impacts of the funding source, empirical studies usually rely on total health expenditure per capita as input indicator for efficiency analysis [12, 16, 24].

In addition to economic and social influence factors, some studies examined the role of governance. There is some evidence that countries with better rule of law have more efficient health systems [14]. The results of Jordi et al. indicate that governance, defined as institutional strength, institutional memory and political commitment, has a strong positive impact on efficiency [15]. Those findings of governance-efficiency relationships are in line with the more general positive impacts of good governance on population health reported in the empirical literature [29–31].

In addition to good governance, several studies find evidence that democratic political regimes achieve higher levels of population health than autocratic regimes [32–34]. The main argument for positive impacts of democracy on population health provided by the public choice literature is that democratic governments are more reliant on public support than autocratic governments. As a result, democratic governments may have a higher incentive to provide goods, including healthcare, to generate support in the population [19, 20]. In this regard, Wigley and Akkoyunlu-Wigley argue that the effect of democracy on population health involves both redistributive and non-redistributive channels [35]. However, some empirical findings contradict the hypothesis of a “democratic advantage” [36, 37]. An explanation for this conflicting evidence is provided by Roessler, whose results indicate that democracy has a positive impact on the public provision of goods in countries with sufficiently high income levels only [22].

As Geloso et al. point out, non-democratic leaders may have strong incentives to promote population health outcomes but often fail to deal with trade-offs related to lacks of economic and political freedom [38]. In the context of the COVID-19 pandemic, there is extensive scientific debate on dis-/advantages of different political regime types regarding effective political responses [39–41].

While most studies support the view that democracies achieve better health outcomes, evidence on the impact of democracy on health expenditure is inconclusive [36, 42–44]. Given positive associations between democracy and health outcomes, the absence of a clear link between political regimes and health spending seems puzzling. In this study, we show that one explanation for these seemingly inconsistent results may be obtained by linking democracy to health system efficiency. In particular, our results indicate that democracy affects expenditure on and efficiency of the health system differently.

Our theoretical approach is closely linked to previous models investigating the relationships between corruption and political institutions [45–48]. Although the economic growth model presented by Rivera-Batiz is not focused on the health system, there is some structural similarity to our approach [49]. In this model, democratic institutions are shown to improve governance by constraining the actions of corrupt officials. This, in turn, leads to enhancement of total factor productivity (TFP) as a main determinant of long-run growth. Our model is also closely related to the selectorate theory developed by Bueno de Mesquita et al. [21]. Selectorate theory explains a wide range of political decisions and phenomena, including the provision of public goods, based on the sizes of two groups: 1) the selectorate, defined as the number of people eligible to select the ruler, 2) the winning coalition, defined das the number of supporters a leader needs to remain in power. If the winning coalition is small, it is more beneficial for the leader to provide private goods to members of the winning coalition than to invest in public goods. In case of a large winning coalition, public goods provision is more attractive. Given that democracies are characterized by relatively large winning coalitions, this logic implies that pubic goods provision is higher in democracies than in autocracies. Since freedom from corruption shares some characteristics of a public good, selectorate theory predicts that corruption is more prevalent in autocratic regimes with small winning coalitions [21, 50].

In the following, we draw on the results of studies outlined above to develop a simple theoretical model of government investments in population health. The model combines insights from the literature on health system efficiency and the public choice literature and offers consistent implications for effects of democracy on health outcomes, health spending, and health system efficiency.

## 3 The model

**Fig 1:**
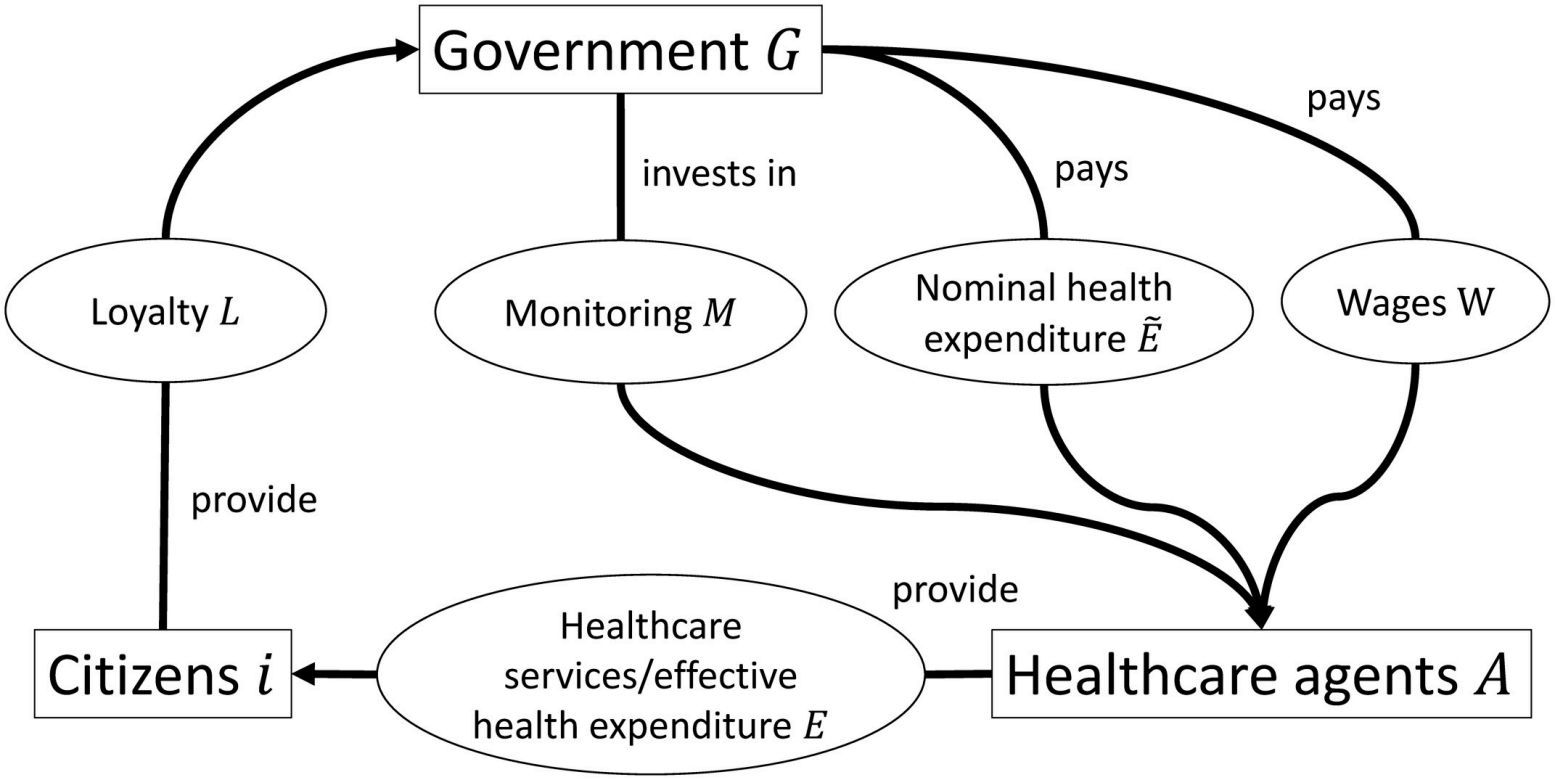
Model structure.

Our model has a simple structure (Fig 1). We consider a government *G*, whose primary aim is to survive in office. To achieve this aim, the government must ensure public support. In the model, public support is represented by the population’s level of loyalty *L* ∈ [0, 1] towards the government. To generate loyalty, the government can invest a certain amount $\tilde{E}\geq 0$ in the provision of healthcare goods and services. In the following, we refer to $\tilde{E}$ as *nominal* health expenditure. For provision of healthcare, the government concludes contracts with healthcare agents *A*. Each of these agents receives a part of nominal health expenditure and a wage *w* ≥ 0, with total wage payments adding up to *W*. A central characteristic of our model is that it considers the fact that some agents may have an incentive to embezzle health expenditure for private gain instead of providing healthcare to the population. Hence, only the amount $E\in [0,\tilde{E}]$ of nominal health expenditure $\tilde{E}$ is used for effective provision of healthcare. We therefore refer to *E* as *effective* health expenditure. To curb embezzlement of health expenditure by the agents, the government can invest in monitoring *M*. As shown in the following, higher monitoring efforts generally reduce incentives for embezzlement and, thus, affect the share of nominal health expenditure $\tilde{E}$ converted to effective health expenditure *E*. Given the mentioned instruments, we derive the optimal policy choice of the government and investigate its implications for health system efficiency.

### 3.1 Model setup

From the perspective of the government, investments in population health $\tilde{E}$, the sum of wage payments to healthcare agents *W* and costs of monitoring *M* reduce budget that could be used for other purposes, including consumption of government officials, vanity projects, or other policy objectives. The government therefore has an incentive to keep these costs at a minimum. We express these preferences by specifying the loss function of the government as

$$\begin{array}{c} V_{G}=\tilde{E}+W+M. \end{array}$$ (1)

Since the government’s main objective is to stay in office, minimization of Eq 1 is subject to the condition that the population’s loyalty *L* is high enough to survive in office. Following Roessler, we impose that the government must reach a certain threshold level of loyalty $\bar{L}\in \left[0,1\right]$ to stay in office [22]. Hence, the government survives in office if $L\geq \bar{L}$ and is removed otherwise. In line with the public choice literature, we further assume that democratic governments must generate a higher level of loyalty to stay in office than non-democratic governments [19, 20]. The threshold level of loyalty therefore is related to the level of democracy *D* ∈ [0, 1] by $\bar{L}=\bar{L}\left(D\right)$, where ${\bar{L}}^{\prime }\left(D\right)>0,\bar{L}\left(0\right)>0$, and $\bar{L}\left(1\right)<1$. The positive derivative ${\bar{L}}^{\prime }\left(D\right)>0$ implies that the threshold level of loyalty increases in the level of democracy, which reflects that democratic governments must generate more public support. Furthermore, also fully autocratic governments (*D* = 0) must ensure a certain level of loyalty ($\bar{L}\left(0\right)>0$) in the population as they face the threat of revolution [51].

The population consists of a continuum citizens *i* ∈ [0, 1] with mass normalized to unity. The loyalty of each citizen is determined by the relation between her status-quo utility *U*_*i*_ and an individual-specific threshold utility ${\bar{U}}_{i}$. A citizen is assumed to support the government only if $U_{i}\geq {\bar{U}}_{i}$. Since our model focuses on provision of healthcare, we express status-quo utility as a function of the citizen’s health *h*_*i*_ > 0, i.e. *U*_*i*_ = *U*(*h*_*i*_), with *U*′(*h*_*i*_) > 0. The citizen’s health, in turn, is described by the micro-level production function

$$\begin{array}{c} h_{i}=\varphi \cdot E^{\alpha }\cdot \psi_{i} \end{array}$$ (2)

where *φ* > 0 is a productivity parameter, *ψ*_*i*_ > 0 captures individual-specific determinants of health, *E* is effective health expenditure and *α* ∈ (0, 1) is the elasticity of individual health with respect to effective health expenditure. Eq 2 thus formalizes that effective provision of healthcare goods and services increases individual health. Normalizing *ψ*_*i*_ to unity, the macro-level health production function can be derived from Eq 2 by aggregating over all individuals:

$$\begin{array}{c} H=\int_{0}^{1}h_{i}\;\text{d}i=\varphi \cdot E^{\alpha }. \end{array}$$ (3)

In the following, we refer to *H* as population health. Given these expressions, the citizen’s condition for loyalty towards the government can be written as

$$\begin{array}{c} \varphi \cdot E^{\alpha }=H\geq \xi_{i}, \end{array}$$ (4)

where $\xi_{i}≔U^{-1}\left({\bar{U}}_{i}\right)/\psi_{i}$ captures the citizen’s predisposition for reaching the loyalty threshold and may be interpreted as capturing the individual-specific propensity to support the government. Denoting the density function of *ξ*_*i*_ by *f*(*ξ*), with *f*′(*ξ*)> 0∀*ξ* > 0, the population’s level of loyalty towards the government can be derived from Eq 4 as

$$\begin{array}{c} L=L\left(H\right)=\int_{0}^{H}f\left(\xi \right)\;\text{d}\xi . \end{array}$$ (5)

From Eq 5 follows that *L*′(*H*) = *f*(*H*) > 0, i.e. increases in population health are related to a higher level of loyalty. This reflects that better health induces a higher status-quo utility of the citizens, which, in turn, promotes public support for the government.

Having derived the population’s level of loyalty, we can formulate the problem of the government as

$$\begin{array}{c} \text{min}\;V_{G}=\tilde{E}+W+M\;\text{s}.\text{t}.\;L\left(H\right)\geq \bar{L}\left(D\right), \end{array}$$ (6)

which implies that the government minimizes health-related costs while ensuring that population health *H* is high enough to reach the loyalty threshold determined by the level of democracy *D*.

To produce population health, the government concludes contracts with healthcare agents *A*. Each of these agents receives one unit of *nominal* health expenditure, i.e. $\tilde{e}=1$, to provide healthcare to the citizens. The total mass of agents therefore is equal to nominal health expenditure $\tilde{E}$. As compensation for provision of healthcare, each agent also receives a wage *w*. Given *w* and $\tilde{e}$, each agent has two options: 1) The agent can contribute to effective healthcare with the amount of *e* = 1 and consume her wage *w*. 2) The agent can consume both, $\tilde{e}$ and *w*, and make no contribution to effective healthcare, i.e. *e* = 0.

In case of choosing option 1), the agent’s utility is given by $U_{A}^{h}=w\cdot {\bar{\varepsilon }}_{A}$, where ${\bar{\varepsilon }}_{A}\geq 1$ reflects the agents intrinsic motivation to behave in line with the contract and contribute to effective healthcare. Hence, ${\bar{\varepsilon }}_{A}$ may be referred to as the agent’s integrity.

In case of choosing option 2), the agent draws utility from consumption of *w* and $\tilde{e}$ only if she is not convicted of embezzlement by the government. Otherwise, she receives a payoff of 0. The probability of being convicted is related to the monitoring efforts *M* of the government. We let *p* = *p*(*M*) ∈ [0, 1] denote the probability that a corrupt agent is convicted, with *p*′(*M*) > 0 and *p*′′(*M*) < 0 capturing that higher monitoring efforts of the government increase the probability of being convicted but at a decreasing rate. The expected utility of the agent when choosing option 2) therefore is $U_{A}^{0}=\left[1-p\left(M\right)\right]\cdot \left(w+\tilde{e}\right)=\left[1-p\left(M\right)\right]\cdot \left(w+1\right)$.

Given these payoffs, the agent chooses option 1) and contributes to healthcare only if $U_{A}^{H}\geq U_{A}^{0}$. This condition may be written as

$$\begin{array}{c} \varepsilon_{A}\leq \frac{w}{w+1}\cdot \frac{1}{1-p(M)}, \end{array}$$ (7)

where $\varepsilon_{A}=1/\bar{\varepsilon_{A}}\in \left[0,1\right]$ reflects the agent’s propensity for corruption. Assuming that *ε*_*A*_ is uniformly distributed over [0, 1], Eq 7 implies that the amount of effective health expenditure is given by For simplicity, we focus on interior solutions only, i.e. $E\in (0,\tilde{E})$.

$$\begin{array}{c} E=\frac{w}{w+1}\cdot \frac{1}{1-p(M)}\cdot \tilde{E}. \end{array}$$ (8)

Eq 8 links effective health expenditure *E* to nominal health expenditure $\tilde{E}$. The share of nominal health expenditure converted to effective health expenditure is positively related to wage *w*. This reflects that a higher wage leads to a stronger increase in utility from behaving in accordance with the contract than in expected utility from embezzlement. Hence, increasing *w* decreases the share of corrupt agents and induces higher effective health expenditure. Furthermore, higher monitoring efforts *M* of the government lead to higher effective health expenditure as they increase the corrupt agents’ probability *p*(*M*) of being convicted. Following Eq 7, this induces a higher share of agents providing healthcare.

### 3.2 Equilibrium

Since production of population health is costly, Eq 6 implies that the government will not generate more loyalty than required to stay in office in order to save costs, i.e. $L\left(H\right)=\bar{L}\left(D\right)$. Using Eqs 3 and 8, nominal health expenditure therefore can be expressed as

$$\begin{array}{c} \tilde{E}=\frac{w+1}{w}\cdot \left[1-p\left(M\right)\right]\cdot {\left(\frac{\vartheta (D)}{\varphi }\right)}^{1/\alpha }, \end{array}$$ (9)

where $\vartheta \left(D\right)=L^{-1}\left(\bar{L}\left(D\right)\right)$. Since the properties of *L*(⋅) and $\bar{L}\left(\cdot \right)$ imply that *ϑ*′(*D*) > 0, higher levels of democracy generally increase the government’s incentive to increase nominal health expenditure. However, expression Eq 9 also shows that increases in *D* may be compensated by changes in the wage of healthcare agents *w* and monitoring efforts *M*. To determine *w* and *M*, we use Eq 9 and the fact that total wage payments are $W=w\cdot \tilde{E}$ to formulate the government’s problem Eq 6 as

$$\begin{array}{c} \min_{w,M}V_{G}=\frac{{\left(w+1\right)}^{2}}{w}\cdot \left[1-p\left(M\right)\right]\cdot {\left(\frac{\vartheta (D)}{\varphi }\right)}^{1/\alpha }+M \end{array}$$ (10)

The first order conditions following from Eq 10 are

$$\begin{array}{c} w^{*}=\tilde{e}=1, \end{array}$$ (11)

$$\begin{array}{c} \frac{{\left(w^{*}+1\right)}^{2}}{w^{*}}\cdot {\left(\frac{\vartheta (D)}{\varphi }\right)}^{1/\alpha }\cdot p^{\prime }\left(M^{*}\right)=1. \end{array}$$ (12)

According to Eq 11, the equilibrium wage w* is proportional to the nominal health expenditure $\tilde{e}$ received by each agent. This reflects that a higher $\tilde{e}$ increases the agents’ incentives to consume both *w* and $\tilde{e}$ instead of contributing to population health. To counterbalance this increased threat of embezzlement, the government increases the wage to make fulfillment of condition Eq 7 for effective provision of healthcare more likely.

Eq 12 states that the government weighs up the benefits and costs of monitoring *M*. On the one hand, higher monitoring efforts decrease the agents’ incentives for embezzlement and, thus, increase effective health expenditure according to Eq 8. On the other hand, higher monitoring efforts reduce the budget of the government and, hence, increase the value of the loss function Eq 10. The government therefore chooses the optimal monitoring effort M* by equating the marginal gain and the marginal cost of monitoring.

The first order condition Eq 12 further implies that the government’s monitoring efforts depend on the level of democracy *D*. Implicit differentiation of Eq 12 reveals that

$$\begin{array}{c} \frac{\text{d}M^{*}}{\text{d}D}=-\frac{1}{\alpha }\cdot \frac{\vartheta^{\prime }\left(D\right)\cdot p^{\prime }\left(M^{*}\right)}{\vartheta \left(D\right)\cdot p^{\prime \prime }\left(M^{*}\right)}>0. \end{array}$$ (13)

A higher level of democracy induces a higher level of population health that must be ensured by the government to stay in office. As shown by Eq 3, this implies that the government must increase effective health expenditure *E*. One way to achieve this aim is to reduce the loss of nominal healthcare expenditure $\tilde{E}$ due to embezzlement as implied by Eq 8. The government reduces this loss by increasing monitoring efforts M*, which decrease the healthcare agents’ incentives for embezzlement according to Eq 13.

In addition to better monitoring of agents, the government can reach higher levels of effective health expenditure *E* by choosing higher nominal health expenditure $\tilde{E}$. To investigate the impact of democracy *D* on nominal health expenditure $\tilde{E}$, we use Eqs 9 and 13 to derive the marginal effect of democracy:

$$\begin{array}{c} \frac{\text{d}{\tilde{E}}^{*}}{\text{d}D}=\frac{2}{\alpha }\cdot {\left(\frac{\vartheta (D)}{\varphi }\right)}^{1/\alpha }\frac{\vartheta^{\prime }\left(D\right)}{\vartheta (D)}\cdot (\frac{{\left[p^{\prime }\left(M^{*}\right)\right]}^{2}}{p^{\prime \prime }\left(M^{*}\right)}+\left[1-p\left(M^{*}\right)\right])⪌0. \end{array}$$ (14)

It is noteworthy that the sign of Eq 14 is ambiguous, which implies that democracy may have a positive or negative (or no) impact on the nominal amount of health expenditure. Democracy decreases nominal health expenditure if the marginal effect of monitoring on the agents’ probability of being convicted *p*′(M*) is relatively large. In this case, it is beneficial for the government to shift resources from nominal investments in population health to increased monitoring of healthcare agents in order to raise effective health expenditure E*. In contrast, higher levels of democracy induce higher nominal health expenditure ${\tilde{E}}^{*}$ if the marginal effect of monitoring *p*′(M*) is relatively small. These ambiguous results provide an explanation for the inconclusive evidence on the relationship between health expenditure and democracy reported in previous studies.

### 3.3 Implications for health system efficiency

For given resource inputs, efficiency may be assessed by comparing the observed output or outcome to the maximum output or outcome that could be produced with the same amount of inputs. In our model, this corresponds to comparing equilibrium population health H* with the level of population health $\tilde{H}=\varphi \cdot {\tilde{E}}^{\alpha }$ that could optimally be produced with total nominal health expenditure $\tilde{E}$. Using Eq 8, technical efficiency *T* of the healthcare system therefore is given by

$$\begin{array}{c} T=\frac{H^{*}}{\tilde{H}}={\left(\frac{E^{*}}{\tilde{E}}\right)}^{\alpha }=\sigma \cdot {\left(\frac{1}{1-p(M^{*})}\right)}^{\alpha }, \end{array}$$ (15)

where *σ* = (1/2)^*α*^. As shown by Eq 15, health system efficiency depends on the monitoring efforts of the government, which determine the healthcare agents’ incentives for theft and, hence, the loss of nominal health expenditure due to embezzlement. Monitoring efforts, in turn, were shown to depend on the level of democracy according to Eq 13. Calculating the marginal effect of democracy on technical efficiency yields:

$$\begin{array}{c} \frac{\text{d}T}{\text{d}D}=\alpha \sigma \cdot \frac{p^{\prime }\left(M^{*}\right)}{{\left[1-p\left(M^{*}\right)\right]}^{1+\alpha }}\cdot \frac{\text{d}M^{*}}{\text{d}D}>0. \end{array}$$ (16)

Since a higher level of democracy induces higher monitoring efforts of the government (dM*/d*D* > 0), the prevalence of corrupt healthcare agents declines if *D* increases. As a result, a higher share of nominal health expenditure is used for effective provision of healthcare, which is reflected in higher technical efficiency *T*. Thus, higher levels of democracy are related to increased health system efficiency because of reduced embezzlement of health expenditure. Based on this result, we derive the following hypothesis from the theoretical model for empirical examination:

Hypothesis: Higher levels of democracy are related to higher levels of health system efficiency.

## 4 Empirical evidence

### 4.1 Data

To test the hypothesis derived above empirically, we use data on 158 countries over the period 1995–2015. As our main indicator of population health, we use healthy life expectancy (HALE) at birth obtained from the World Health Organization (WHO) [52]. In contrast to “standard” life expectancy, HALE does not only consider length but also quality of life. By capturing various health outcomes, including mortality and disability, HALE represents a broad measure of population health which makes it a suitable indicator for the analysis of health system performance [12, 16, 24]. For robustness checks, we draw on life expectancy at birth and under-5 mortality per 1,000 live births [53] as alternative population health indicators. We use the inverse of under-5 mortality to ensure that higher values indicate better health outcomes.

In line with the theoretical model and the majority of previous studies, we use current health expenditure per capita (in constant 2010 US-Dollar) [53] as indicator for health system inputs. We express health expenditure in per capita terms to account for differences in population size between countries. In addition to the relationship between health outcomes and health expenditure, the link between health and education was explored in previous research [24]. Hence, we use the average years of schooling of the population aged 25+ [54] as additional input in the health production function for sensitivity analysis.

Our main explanatory variable for health system efficiency is democracy. To measure democracy, we draw on the Polity V Project [18], which provides the well-established Polity scores. The Polity scores measure a country’s level of democracy on a scale ranging from -10 (full autocracy) to 10 (full democracy) and, thus, distinguish between different degrees to which political regimes show autocratic and democratic characteristics. For robustness checks, we use three alternative democracy indicators. First, we use the Electoral democracy index (EDI) provided by the V-Dem project [55]. The EDI measures the extent to which the ideal of electoral democracy is reached in a certain country in a specific year on a continuous scale. Second, we use the binary democracy indicator developed by Boix, Miller, and Rosato [56] (in the following referred to as BMR), which only distinguishes between democracies and non-democracies. Democracy measures like the Polity scores, the EDI, and the BMR democracy indicator may be subject to expert bias [57] and measurement error. We therefore also use the binary democracy indicator proposed by Acemoglu et al. [58] (in the following referred to as ANRR), which makes use of information from different data sources to mitigate measurement error. Data on the ANRR democracy indicator are available up to the year 2010 only. Hence, estimations employing this indicator are based on a reduced sample.

While the EDI, the BMR, and the ANRR indicator already have a theoretical range from 0 to 1, we also normalize the Polity scores between 0 (full autocracy) and 1 (full democracy) as Polity^normalized^ = (Polity + 10)/20. This implies that the coefficient of each democracy indicator in the econometric models specified below may be interpreted as the effect of a full-range increase in the level of democracy.

In addition to democracy, the literature highlighted other variables that may influence efficiency of healthcare systems. To control for effects of the countries’ levels of economic development, we include the logarithm of GDP per capita as a covariate in our models. Provision and monitoring of healthcare may be less costly in urban than in rural areas. In line with the literature, we therefore use the share of the population living in urban areas and logged population density as control variables. Countries experiencing violent civil conflict were found to show lower levels of efficiency [24]. Hence, we adjust estimates for the presence of civil conflict as coded by the Uppsala Conflict Data Program [59, 60]. Another factor that may determine population health is air pollution (measured by mean annual exposure to PM2.5 air pollution in micrograms per cubic meter), which also enters our models as a covariate. In addition, there is evidence that the public sector exhibits economies of scale [61]. Our models therefore include the logarithm of the countries’ total population as covariate. Finally, unmeasured regional or cultural factors may influence the efficiency of health systems. Our econometric specifications therefore adjust for the country’s region as defined by the World Bank [53].

Our main indicator of population health, HALE at birth, is available in five-year intervals between 2000 and 2015. We therefore use the average values of per capita health expenditure, the democracy indicators, and the covariates of inefficiency in the preceding five-year periods (1996–2000,…,2011–2015) for empirical estimation. In addition to capturing lagged effects of health expenditure and covariates of inefficiency, calculating period averages has the advantage of mitigating the influence of short-term fluctuations on the results of statistical analyses. Summary statistics for all variables are provided in the appendix (Table A in S1 Appendix).

### 4.2 Method

To derive the econometric specification used for empirical analysis, we use Eqs 3 and 15 to express population health as

$$\begin{array}{c} H^{*}=\varphi \cdot {\left({\tilde{E}}^{*}\right)}^{\alpha }\cdot T. \end{array}$$ (17)

Eq 17 implies that population health may be modeled using observed health expenditure $\tilde{E}$ and technical efficiency *T*. By defining the technical inefficiency *I* = 1/*T*, taking logarithms on both sides of Eq 17, and adding subscripts for country *i* and year *t* and the error term *v*_*it*_, we can derive the econometric model

$$\begin{array}{c} \text{ln}\;H_{it}^{*}=\phi +\alpha \;\text{ln}\;{\tilde{E}}_{it}^{*}-\text{ln}\;I_{it}+v_{it}, \end{array}$$ (18)

where *ϕ* = ln *φ* is a constant. Eq 18 represents a stochastic frontier production function, which links population health to observed health expenditure and inefficiency. This production function provides the basis for stochastic frontier analysis (SFA). Based on our theoretical model, we may further specify the SFA model as follows. Since technical efficiency is hypothesized to depend on democracy *D*, this is also true for inefficiency *I*. The theoretical model does not provide a specification of the inefficiency model. We therefore approximate the relationships between inefficiency, democracy, and other covariates by

$$\begin{array}{c} \text{ln}\;I_{it}=\beta_{0}+\beta_{1}D_{it}+z_{it}^{\prime }\gamma +u_{it}, \end{array}$$ (19)

where ***z*** is the matrix of confounders with coefficients ***γ*** and *u*_*it*_ is a random variable, which is assumed to have a truncated normal distribution.

The econometric model defined by the frontier model Eq 18 and the inefficiency model Eq 19 is equivalent to the well-established SFA model for technical inefficiency effects proposed by Battese and Coelli [62]. In contrast to data envelopment analysis (DEA), SFA accounts for the stochastic nature of the inefficiency estimates without requiring second-stage procedures involving bootstrapping [63]. Moreover, compared to DEA, SFA potentially offers the advantage of consistency and higher statistical efficiency. Following these arguments and for better correspondence and interpretability with regard to the theoretical model, our empirical analysis relies on SFA.

In Eq 19, the coefficient *β*_1_ represents the effect of democracy on inefficiency. Calculating the differential of Eq 19 with respect to *D*_*it*_ yields

$$\begin{array}{c} \frac{\text{d}I_{it}}{I_{it}}=-\frac{\text{d}T_{it}}{T_{it}}=\beta_{1}\cdot \text{d}D_{it}. \end{array}$$ (20)

Given that *D*_*it*_ is normalized between 0 and 1, *β*_1_ ⋅ 100 thus approximates the percentage change in inefficiency due to a full-range increase in the level of democracy (d*D*_*it*_ = 1), e.g. a change from full autocracy to full democracy on the Polity scale.

### 4.3 Baseline results

The estimation results for the baseline SFA model indicate that an increase in per capita health expenditure of 100% is, on average, related to an increase in HALE at birth of 3.4% (Table 1). In line with the theoretical model, the coefficient of the Polity score in the inefficiency model is negative and significant at the 1% level. The estimated efficiency gain of full democracies relative to full autocracies is approximately 11% (*β*_1_ = −0.112, 95%-CI = [−0.174, −0.050]). This result indicates substantial effects of the political regime on health system efficiency. Furthermore, inefficiency is found to be lower in countries with a higher degree of urbanization and higher population density. We do not find significant associations between inefficiency and GDP/capita, internal conflict, PM2.5 air pollution, and total population.

**Table 1:** Baseline SFA results.

Model part	Dependent variable	HALE at birth, log.	
	Variable	Estimate	95%-CI
Frontier model	Health expend./capita, log.	0.034***	(0.031, 0.037)
	Constant	4.004***	(3.981, 4.026)
Inefficiency model	Polity score	-0.112***	(-0.174,-0.050)
	GDP/capita, log.	-0.017	(-0.038, 0.004)
	Urban population, share	-0.299***	(-0.427,-0.170)
	Population density, log.	-0.040***	(-0.052,-0.028)
	Internal conflict	-0.007	(-0.061, 0.047)
	PM2.5 air pollution, log.	-0.021	(-0.064, 0.023)
	Population, log.	-0.005	(-0.016, 0.006)
	Constant	0.469***	(0.195, 0.744)
Countries		158	
Observations		615	

Significance levels:

*10%,

**5%,

***1%;

log. = logarithm

**Fig 2:**
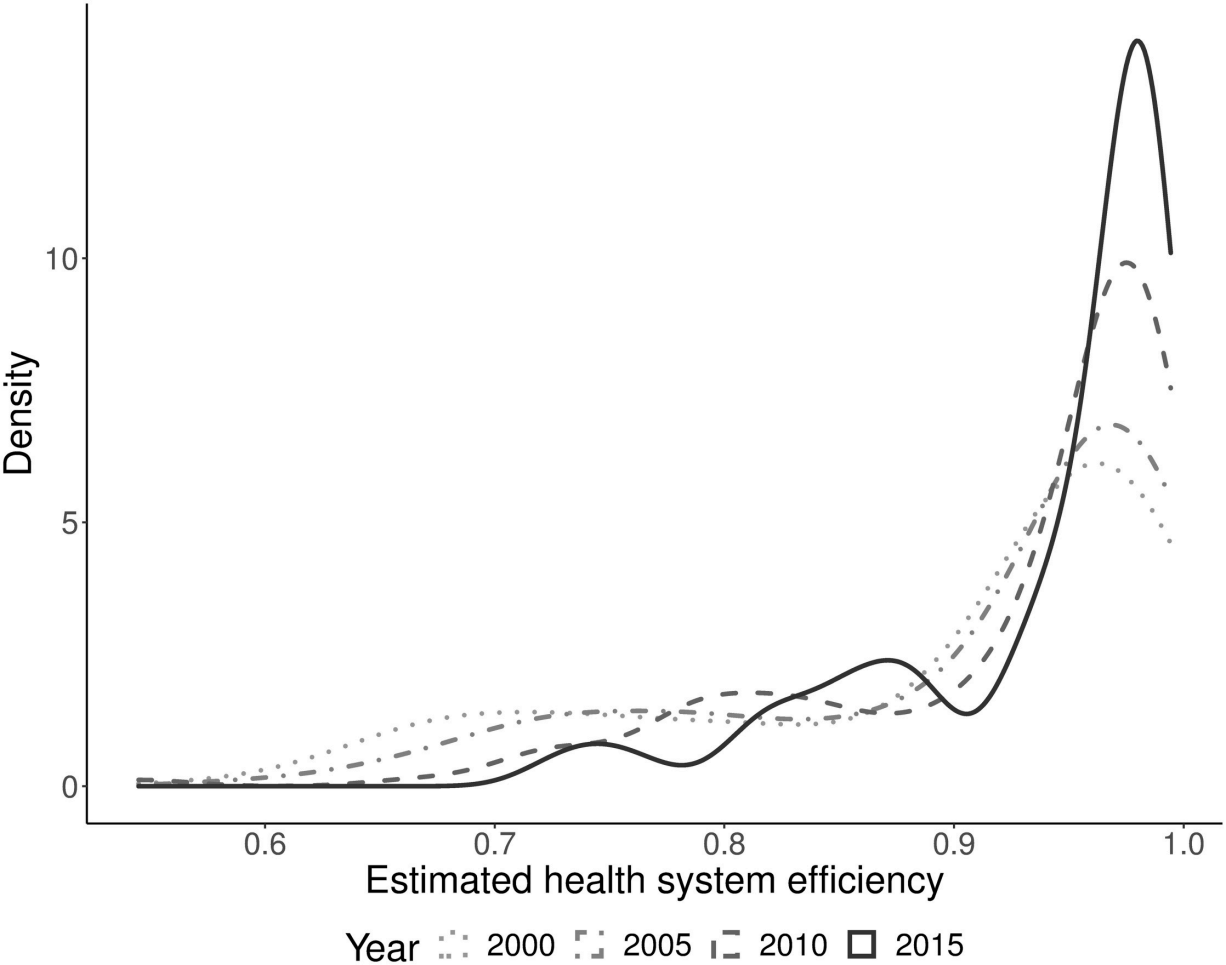
Distribution of estimated health system efficiency of 149 countries over time. Note: To ensure that changes in the distribution of inefficiency estimates over time are not driven by changes in the composition of countries, the depicted data include only countries for which efficiency scores could be derived for all years. The full set of 158 countries was included in estimation of the SFA model.

The distribution of health system efficiency derived from the baseline SFA indicates improvements in efficiency over time (Fig 2). Average health system efficiency increased from 0.89 in 2000 to 0.94 in 2015. However, 25% of the countries in 2015 had efficiency scores of approximately 0.9 or lower, with a minimum of 0.72. This implies substantial losses in production of population health.

**Fig 3:**
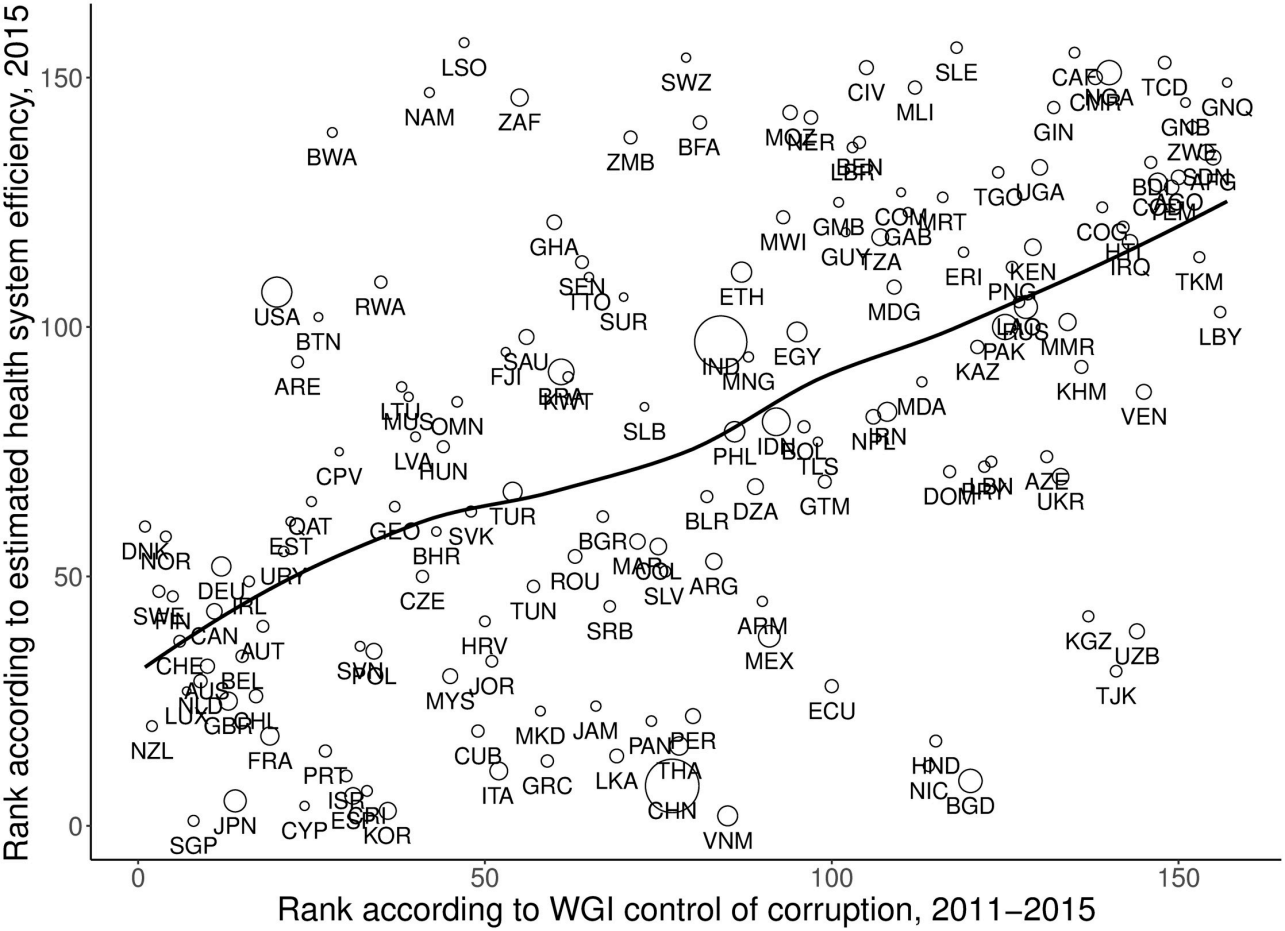
Relationship between ranking of countries according to estimated health system efficiency and control of corruption (n = 157). Note: The figure shows only countries with available data on both health system efficiency and control of corruption (*n* = 157). The full set of 158 countries was included in estimation of the SFA model. The sizes of the circles represent total population size.

The theoretical model postulates that the scope for embezzlement of health expenditure is a main channel through which democracy may influence health system efficiency. Relating “Control of corruption” provided by the Worldwide Governance Indicators (WGI) project as a broad indicator of government efforts to prevent embezzlement to estimated health system efficiency supports this assumption (Fig 3). Countries ranking high in terms of control of corruption tend to rank high in terms of health system efficiency and vice versa (rank correlation: *ρ* = 0.54, *p* < 0.01). Corruption, in turn, is lower in countries with higher Polity scores (*ρ* = 0.57, *p* < 0.01). In addition, the WGI control of corruption indicator is closely related to government efforts to prevent corruption as measured by the indicator “Anti-corruption policy” provided by the Bertelsmann Transformation Index [64] for developing and emerging markets countries (see Figure A in S1 Appendix). The anti-corruption policy indicator measures whether “adequate institutional arrangements exist to implement an anti-corruption policy and if they successfully contribute to an effective prosecution of corruption” [64]. This indicator is also positively correlated with the level of democracy as measured by the Polity scores (*ρ* = 0.53, *p* < 0.01), which provides further indirect support for the main mechanism of the theoretical model.

The relationship between health system efficiency and democracy also becomes obvious when grouping countries into political regimes as proposed by the Polity project [18]. Distinguishing between autocracies (Polity scores between -10 and -6), anocracies (Polity scores between -5 and 5), and democracies (Polity scores between 6 and 10) reveals that countries belonging to the same regime type tend to have more similar efficiency scores than countries belonging to different regime types (Figure B in S1 Appendix).

### 4.4 Robustness checks

#### 4.4.1 Results for alternative democracy indicators

Replacing the Polity scores by the V-Dem EDI, the binary BMR democracy indicator, and the ANRR democracy indicator, respectively, does not change SFA results qualitatively (Table 2). The coefficients of all democracy indicators are negative and statistically significant, indicating that democracies have more efficient health systems than non-democracies.

**Table 2:** SFA results for alternative democracy indicators.

Dependent variable	HALE at birth, log.		HALE at birth, log.		HALE at birth, log.	
Variable	Estimate					
Health expend./capita, log.	0.034***	(0.030, 0.037)	0.034***	(0.031, 0.037)	0.031***	(0.026, 0.035)
Constant	4.006***	(3.983, 4.029)	4.005***	(3.983, 4.028)	4.025***	(3.993, 4.056)
VDem EDI	-0.156***	(-0.240,-0.071)				
BMR democracy			-0.063***	(-0.097,-0.029)		
ANRR democracy					-0.049**	(-0.087,-0.011)
GDP/capita, log.	-0.014	(-0.035, 0.007)	-0.016	(-0.036, 0.005)	-0.025**	(-0.050,-0.001)
Urban population, share	-0.286***	(-0.413,-0.159)	-0.301***	(-0.434,-0.168)	-0.257***	(-0.406,-0.107)
Population density, log.	-0.041***	(-0.053,-0.029)	-0.039***	(-0.051,-0.027)	-0.038***	(-0.052,-0.024)
Internal conflict	-0.019	(-0.073, 0.035)	-0.010	(-0.069, 0.048)	-0.008	(-0.065, 0.048)
PM2.5 air pollution, log.	-0.015	(-0.056, 0.026)	-0.006	(-0.046, 0.035)	-0.007	(-0.053, 0.039)
Population, log.	-0.004	(-0.015, 0.007)	-0.007	(-0.018, 0.005)	-0.001	(-0.013, 0.011)
Constant	0.422***	(0.148, 0.697)	0.408***	(0.135, 0.680)	0.430***	(0.125, 0.736)
	158		156		151	
	618		615		446	

Significance levels:

*10%,

**5%,

***1%;

log. = logarithm

#### 4.4.2 Results for alternative population health indicators

Using life expectancy at birth and the inverted under-5 mortality rate, respectively, as alternative population health indicators does not change evidence on effects of democracy on health system efficiency (Table 3). In both SFA models, higher Polity sores are significantly related to lower inefficiency.

**Table 3:** SFA results for alternative population health indicators.

Model part	Dependent variable	Life expectancy, log.		Under-5 mortality, inv. log.	
	Variable	Estimate	95%-CI	Estimate	95%-CI
Frontier model	Health expend./capita, log.	0.033***	(0.030, 0.036)	0.251***	(0.159, 0.344)
	Constant	4.127***	(4.107, 4.148)	-3.233***	(-4.020,-2.446)
Inefficiency model	Polity score	-0.111***	(-0.173,-0.049)	-0.292***	(-0.435,-0.148)
	GDP/capita, log.	-0.012	(-0.034, 0.011)	-0.177***	(-0.290,-0.064)
	Urban population, share	-0.291***	(-0.425,-0.157)	-0.517***	(-0.847,-0.188)
	Population density, log.	-0.042***	(-0.054,-0.029)	-0.115***	(-0.143,-0.087)
	Internal conflict	0.000	(-0.058, 0.058)	0.057	(-0.072, 0.186)
	PM2.5 air pollution, log.	-0.034	(-0.081, 0.014)	0.131**	(0.030, 0.233)
	Population, log.	-0.002	(-0.014, 0.010)	0.005	(-0.024, 0.033)
	Constant	0.479***	(0.163, 0.794)	2.901***	(1.597, 4.205)
Countries		158		158	
Observations		615		615	

Significance levels:

*10%,

**5%,

***1%;

log. = logarithm

#### 4.4.3 Results for additional inputs of the frontier model

The frontier model Eq 18 includes per capita health expenditure as the only input to the production of population health. However, population health may also be related to the educational attainment of the population [24]. In addition, medical progress may lead to better health outcomes for a given amount of health expenditure. We model these aspects by specifying the extended frontier model

$$\begin{array}{c} \text{ln}\;H_{it}^{*}=\phi +\alpha \;\text{ln}\;{\tilde{E}}_{it}^{*}+\eta S_{it}+\lambda t-\text{ln}\;I_{it}+v_{it}, \end{array}$$ (21)

where *S*_*it*_ denotes the population’s average years of schooling with coefficient *η*. The coefficient λ of time *t* represents Hicks-neutral technological change, which is included in the frontier function to capture effects of medical progress.

SFA based on Eq 21 yields a positive but insignificant coefficient of “Schooling” and a positive and significant coefficient of time *t* (Table 4). These results provide evidence that medial progress affected the production frontier for HALE at birth. Qualitatively, the results of the inefficiency model remain robust against the inclusion of the additional inputs in the frontier model. Compared to the baseline model, the estimated efficiency gain from democracy decreases (in absolute terms) but remains statistically significant. Notably, the number of included countries and observations are reduced in the extended frontier model due to missing values in educational attainment, which may induce changes in effect estimates and reduced statistical power. The distribution of estimated health system efficiency derived from the extended model is similar to the distribution shown by Fig 2 (see Figure C in S1 Appendix).

**Table 4:** SFA results for extended frontier model.

Model part	Dependent variable	HALE at birth, log.	
	Variable	Estimate	95%-CI
Frontier model	Health expend./capita, log.	0.029***	(0.025, 0.033)
	Schooling	0.002	(-0.001, 0.004)
	*t*	0.009***	(0.006, 0.013)
	Constant	3.994***	(3.968, 4.021)
Inefficiency model	Polity score	-0.099***	(-0.163,-0.036)
	GDP/capita, log.	-0.044***	(-0.065,-0.024)
	Urban population, share	-0.234***	(-0.353,-0.115)
	Population density, log.	-0.042***	(-0.054,-0.030)
	Internal conflict	0.007	(-0.044, 0.057)
	PM2.5 air pollution, log.	-0.059***	(-0.100,-0.018)
	Population, log.	-0.009	(-0.021, 0.002)
	Constant	0.902***	(0.611, 1.194)
Countries		131	
Observations		514	

Significance levels:

*10%,

**5%,

***1%;

log. = logarithm

#### 4.4.4 Results for interactions between democracy and income

Following previous evidence [22], democracy may be expected to promote population health particularly in the presence of relatively high income levels. Given that our model implies that democracy affects health through increased health system efficiency, there may be interactions between democracy and per capita income in our inefficiency model. Hence, we include a multiplicative interaction term between democracy and logged GDP per capita in the inefficiency model, which is specified as

$$\begin{array}{c} \text{ln}\;I_{it}=\beta_{0}+\beta_{1}D_{it}+\beta_{2}\;\text{ln}\;y_{it}+\beta_{3}D_{it}\times \text{ln}\;y_{it}+\beta_{4}{\left(\text{ln}\;y_{it}\right)}^{2}+z_{it}^{\prime }\gamma +u_{it}, \end{array}$$ (22)

where *y*_*it*_ denotes GDP per capita. The marginal effect of democracy can be derived from Eq 22 as

$$\begin{array}{c} \frac{\partial \;\text{ln}\;I_{it}}{\partial D_{it}}=\beta_{1}+\beta_{3}\;\text{ln}\;y_{it}, \end{array}$$ (23)

implying that the relationship between inefficiency and democracy depends on income. In addition to the interaction between democracy and income, this model specification includes the square of logged GDP per capita to account for potential nonlinearities in the relationship between health system inefficiency and logged income.

The estimated coefficients of all interaction terms between the democracy indicators and logged GDP per capita are negative and statistically significant, which implies that higher levels of democracy are more strongly related to lower inefficiency in countries with higher income levels (Table 5). It is noteworthy that Eq 23 implies that the marginal effect of democracy on inefficiency may even be positive. According to the results obtained using the Polity scores as democracy indicator, a positive point estimate of the marginal effect was estimated for 10.2% of the observations in our sample (see Figure D in S1 Appendix). This finding is also in line with previous evidence on adverse effects of democracy in countries with low income levels [22]. A significant coefficient of the squared term of logged GDP per capita was found only when using the Polity scores or the ANRR democracy indicator.

**Table 5:** SFA results for interactions between democracy and income.

Dependent variable	HALE at birth, log.		HALE at birth, log.		HALE at birth, log.		HALE at birth, log.	
Variable	Estimate	95%-CI	Estimate	95%-CI	Estimate	95%-CI	Estimate	95%-CI
Health expend./capita, log.	0.032***	(0.029, 0.035)	0.032***	(0.028, 0.035)	0.032***	(0.029, 0.035)	0.029***	(0.024, 0.033)
Constant	4.017***	(3.993, 4.041)	4.018***	(3.993, 4.043)	4.015***	(3.992, 4.038)	4.038***	(4.007, 4.069)
Polity score	0.537***	(0.177, 0.897)						
Polity score X GDP/capita, log.	-0.085***	(-0.133,-0.038)						
VDem EDI			0.637**	(0.120, 1.154)				
VDem EDI X GDP/capita, log.			-0.102***	(-0.169,-0.035)				
BMR democracy					0.264**	(0.055, 0.472)		
BMR democracy X GDP/capita, log.					-0.045***	(-0.074,-0.016)		
ANRR democracy							0.296***	(0.072, 0.521)
ANRR democracy X GDP/capita, log.							-0.048***	(-0.080,-0.017)
GDP/capita, log.	0.173**	(0.015, 0.331)	0.100	(-0.055, 0.256)	0.069	(-0.078, 0.216)	0.142	(-0.028, 0.312)
(GDP/capita, log.)^2^	-0.009*	(-0.019, 0.000)	-0.005	(-0.015, 0.005)	-0.005	(-0.014, 0.005)	-0.009*	(-0.020, 0.002)
Urban population, share	-0.282***	(-0.405,-0.158)	-0.270***	(-0.403,-0.137)	-0.288***	(-0.414,-0.162)	-0.268***	(-0.415,-0.120)
Population density, log.	-0.038***	(-0.050,-0.026)	-0.040***	(-0.053,-0.027)	-0.037***	(-0.049,-0.025)	-0.035***	(-0.048,-0.022)
Internal conflict	0.022	(-0.032, 0.076)	0.012	(-0.045, 0.068)	0.011	(-0.043, 0.065)	0.009	(-0.049, 0.066)
PM2.5 air pollution, log.	-0.024	(-0.066, 0.019)	-0.021	(-0.068, 0.026)	-0.015	(-0.054, 0.025)	-0.014	(-0.060, 0.032)
Population, log.	-0.004	(-0.015, 0.007)	-0.005	(-0.017, 0.007)	-0.005	(-0.016, 0.005)	0.000	(-0.013, 0.012)
Constant	-0.403	(-1.072, 0.267)	-0.116	(-0.771, 0.540)	0.056	(-0.542, 0.655)	-0.256	(-0.957, 0.445)
Countries	158		158		156		151	
Observations	615		618		615		446	

#### 4.4.5 Addressing endogeneity

Our baseline specification treats democracy as an exogenous regressor in the inefficiency model. However, several studies highlight that political institutions may depend on and interact with social and economic development [65–67]. Consequently, our estimations may suffer from endogeneity of democracy, which may result in biased and inconsistent estimators of model coefficients.

To address endogeneity in panel stochastic frontier analysis, we use the approach proposed by Karakaplan and Kutlu [68] as implemented in the user-written Stata package “XTSFKK” [69]. Similar to other instrumental variables approaches, the main challenge when applying this method is to find suitable instruments that are correlated with the endogenous variables and fulfill the exclusion restriction. Regarding democracy, we draw on the work of Acemoglu et al. [58] who build on the observation that democratizations often happen in regional waves. Following their empirical strategy, we use the average Polity scores of countries with the same political regime type (Analogous to Acemoglu et al. [58], and using the thresholds proposed by the Polity project [18], we distinguish between democracies (Polity socre ≥ 6) and non-democracies (Polity score ≤ 5) to form groups of countries with similar regime types) at the start of our sample in the same World Bank region as instrument for the countries’ level of democracy. In a second specification, we also account for the potential endogeneity of logged GDP per capita by using logged natural resources rents per capita from the World Development Indicators [53] as instrument. The idea behind the choice of this instrument is that rents from natural resources may be linked to income through several channels [70] while not directly affecting population health. In addition, rents from natural resources, particularly oil, have been linked to lower levels of democracy [71].

The results of the SFA estimations accounting for endogeneity of democracy are in line with the hypothesis deduced from the theoretical model (Table 6). For all considered population health indicators (HALE at birth, life expectancy, and under-5 mortality), the coefficient of the Polity score is negative and statistically significant. As shown in the table, this result remains stable when additionally accounting for endogeneity of GDP per capita. Accordingly, these estimation results strengthen the evidence for positive effects of democracy on health system efficiency.

**Table 6:** SFA results accounting for endogeneity of democracy and GDP per capita.

Model part	Dependent variable	HALE at birth, log.		Life expectancy, log.		Under-5 mortality, inv. log.	
	Variable	Est. (SE)	Est. (SE)	Est. (SE)	Est. (SE)	Est. (SE)	Est. (SE)
Frontier model	Health expend./capita, log.	0.028***	0.027***	0.030***	0.028***	0.565***	0.635***
		(0.002)	(0.002)	(0.002)	(0.002)	(0.028)	(0.031)
	Constant	1.743***	1.753***	4.151***	4.161***	-5.336***	-5.721***
		(0.016)	(0.016)	(0.014)	(0.014)	(0.172)	(0.163)
Inefficiency model	Polity score	-0.639**	-0.527**	-1.151***	-1.038***	-0.795***	-0.728***
		(0.252)	(0.246)	(0.217)	(0.216)	(0.208)	(0.213)
	GDP/capita, log.	-0.505***	-0.711***	-0.307***	-0.440***	0.052	0.258***
		(0.112)	(0.119)	(0.093)	(0.101)	(0.084)	(0.095)
	Urban population, share	-3.234***	-2.729***	-2.398***	-2.129***	-1.196**	-1.843***
		(0.746)	(0.745)	(0.646)	(0.647)	(0.523)	(0.548)
	Population density, log.	-0.711***	-0.745***	-0.752***	-0.767***	-0.376***	-0.397***
		(0.107)	(0.107)	(0.105)	(0.104)	(0.086)	(0.086)
	Internal conflict	0.431***	0.388***	0.473***	0.441***	0.214**	0.194*
		(0.117)	(0.114)	(0.095)	(0.094)	(0.102)	(0.113)
	PM2.5 air pollution, log.	0.077	0.115	-0.441	-0.395	-0.315	-0.183
		(0.297)	(0.298)	(0.271)	(0.271)	(0.235)	(0.242)
	Population, log.	-0.264***	-0.258***	-0.257***	-0.249***	-0.090	-0.102
		(0.088)	(0.089)	(0.085)	(0.085)	(0.073)	(0.073)
	Constant	7.716***	8.975***	7.444***	8.142***	4.372***	2.942**
		(1.649)	(1.658)	(1.470)	(1.470)	(1.370)	(1.451)
Polity endogenous		yes	yes	yes	yes	yes	yes
GDP/capita endogenous			yes		yes		yes
Countries		157	157	157	157	157	157
Observations		611	609	611	609	611	609

Significance levels:

*10%,

**5%,

***1%;

log. = logarithm, SE = standard error. Note: Polity scores are instrumented using regional Polity scores calculated following Acemoglu et al. [58]. Logged GDP per capita ins instrumented using logged natural resources rents per capita.

## 5 Conclusions

While previous studies on determinants of health system efficiency focused primarily on economic and social influence factors, the role of the political regime has been neglected. In addition, there is a lack of formal theoretical models on health system efficiency ensuring transparency and logical consistency of arguments and implications. Against that background, this study provided a rigorous analysis of government behavior with respect to investments in population health and efforts to increase health system efficiency. As a main implication, the model predicts that democratic governments put more effort in reducing inefficiencies than non-democratic governments. Hence, democratic countries are expected to show higher health system efficiency than non-democratic countries.

We tested this implication empirically by applying SFA to a broad dataset of 158 countries over the period 1995–2015. The results support the hypothesis derived from the theoretical model and withstand multiple robustness checks, including the use of alternative democracy indicators, alternative health outcomes, additional inputs in the stochastic frontier model, and adjustment for endogeneity.

From a policy perspective, these results have relevant implications for efforts to foster population health, e.g. by providing development assistance in the form of health aid. Such efforts may be less effective in countries characterized by a high degree of corruption in the health system, which may involve significant embezzlement of health expenditure. In this regard, democratically governed countries with more accountable governments may be more likely to reduce those inefficiencies and make additional inputs to the health system more effective in promoting population health. However, the results of our interaction models indicate that this may only be true for democratic countries reaching a certain level of economic development. In poor countries, democracy may even have adverse effects on health system efficiency. An explanation for this finding proposed by Roessler [22] is that the higher amount of public investment a democratic government must provide to stay in office increases the government’s incentives for kleptocratic behavior. This is particularly the case in countries with low income levels, where the tax base available to the government is small. This argument is reinforced by the observation of Aghion et al. [65], who highlight that richer countries have better functioning fiscal systems.

In addition to fungibility of aid [72], the efficiency of the health system and instruments for its improvement could be considered by potential donors. In this regard, deeper insights into the relationships between democracy and health system efficiency would be valuable for targeted policy recommendations and could be gained e.g. by comprehensive case studies. Based on the insights from our more general theoretical model, these aspects could be investigated in future studies on political institutions and health system efficiency. In addition, also non-altruistic donors should consider potential positive spill-over effects of strengthening even inefficient health systems, e.g. in the context of the COVID-19 pandemic. Such transnational effects were not captured by our model and would be another interesting route for further research.

## Supporting information

S1 Appendix Additional results. (PDF)
