# Supplementary material for: Health system efficiency and democracy: A public choice perspective
Source: PLoS One. 2021 Sep 7;16(9):e0256737. doi: 10.1371/journal.pone.0256737 (PMC8423257; doi:10.1371/journal.pone.0256737)
Supplement: S1 Appendix — (PDF) [file pone.0256737.s001.pdf]

Supplementary Material
Health system efficiency and democracy: A public choice perspective

Martin Roessler[1*], Jochen Schmitt[1]

1 Zentrum für Evidenzbasierte Gesundheitsversorgung, Universitätsklinikum und Medizinische Fakultät Carl Gustav Carus an der Technischen Universität Dresden, Dresden, Germany

* martin.roessler@uniklinikum-dresden.de

Table A. Summary statistics

Variable	N	Mean	SD
HALE at birth	621	60.42	(8.66)
Life expectancy	621	67.88	(9.88)
Under-5 mortality	621	45.52	(46.91)
Health expenditure per capita	621	874.3	(1564.24)
Average years of schooling	519	7.21	(3.16)
Polity 2	618	3.6	(6.27)
V-Dem EDI	621	0.53	(0.26)
BMR democracy	618	0.55	(0.49)
ANRR democracy	449	0.64	(0.46)
GDP per capita	621	12002.15	(18092.52)
Urban population	621	0.55	(0.23)
Internal conflict	621	0.13	(0.3)
PM2.5 air pollution	621	30.11	(17.63)
Population density	618	156.4	(549)
Population (in 1,000)	621	41364.2107	(142885.23)

Note: SD = standard deviation

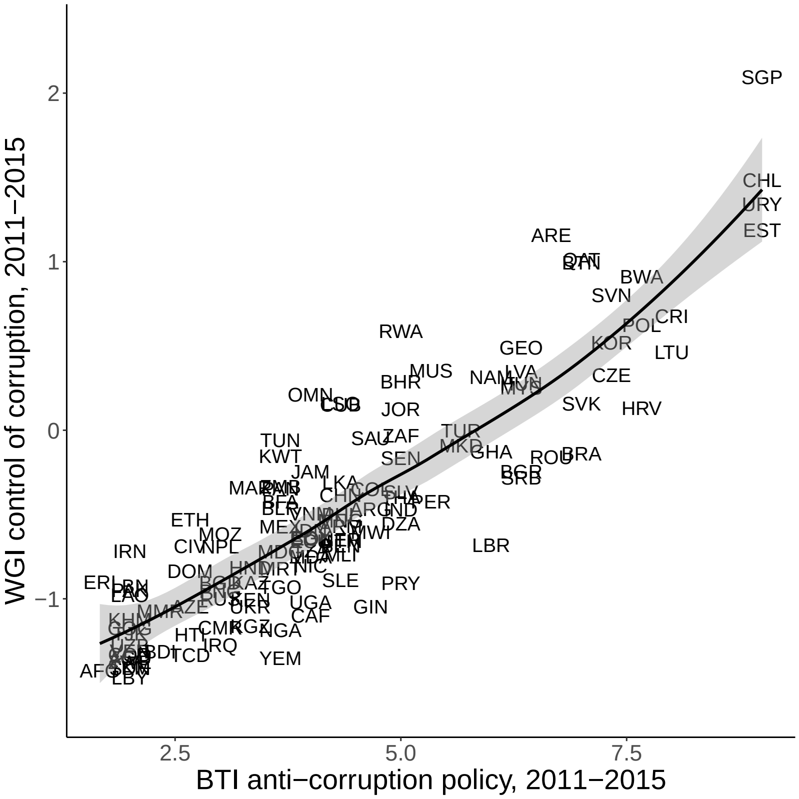

Fig A. Relationship between BTI anti-corruption policy and WGI control of corruption with loess-fit and 95%-confidence interval (n=120)

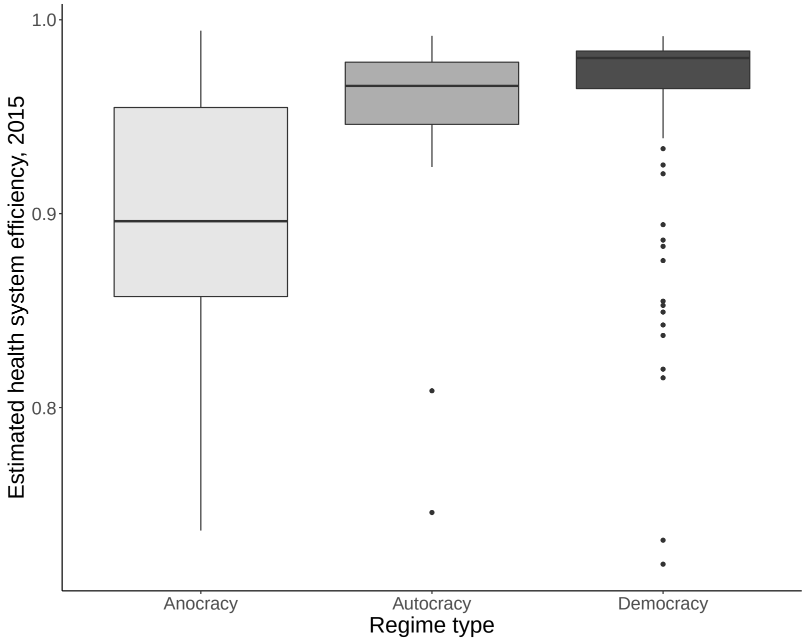

Fig B. Distribution of estimated health system efficiency by regime type
Note: Autocracies (n=19): Polity score between -10 and -6; Anocracies (n=49): Polity score between -5 and 5; Democracies (n=89): Polity score between 6 and 10.

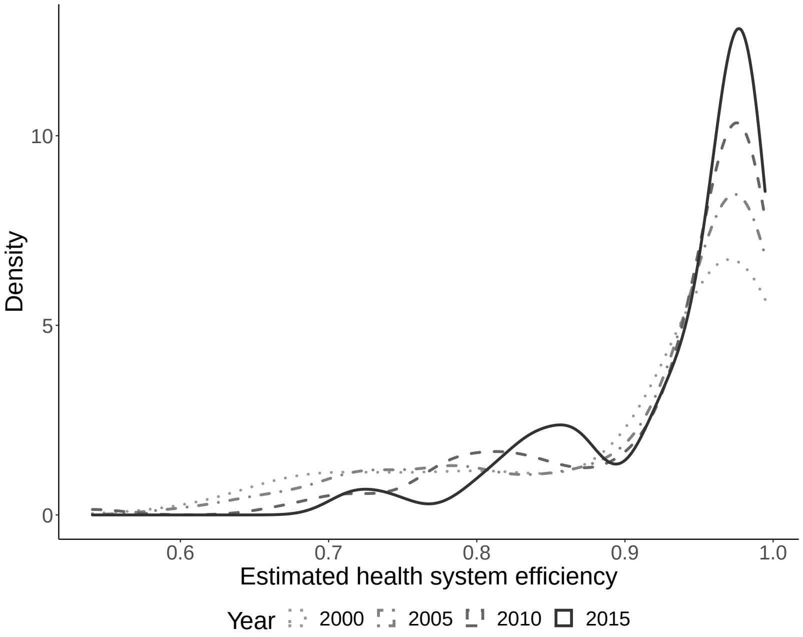

Fig C. Distribution of estimated health system efficiency of 125 countries over time derived from the extended frontier model

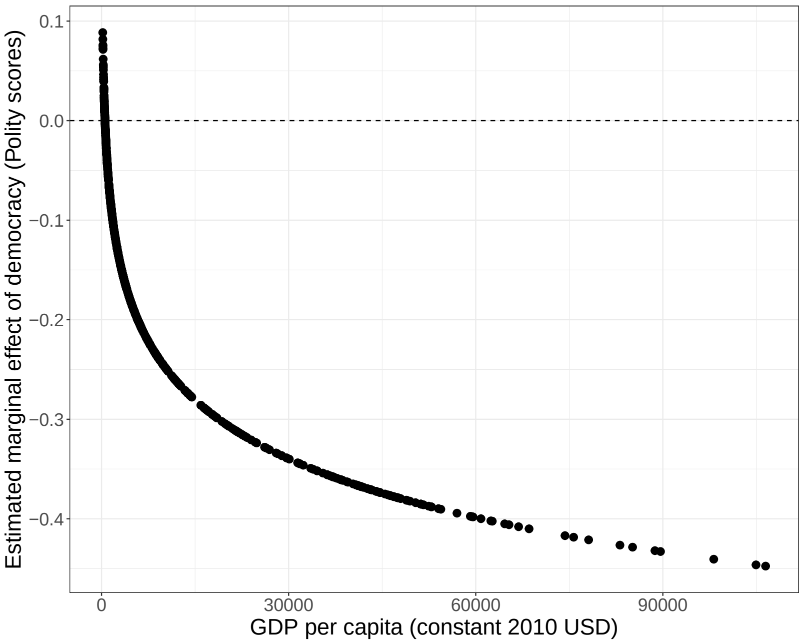

Fig D. Estimated marginal effects of democracy as measured by the Polity scores on inefficiency (logged) derived from the interaction model for the whole sample (n=625)
